# Synergy Pattern of Short Cationic Antimicrobial Peptides Against Multidrug-Resistant *Pseudomonas aeruginosa*

**DOI:** 10.3389/fmicb.2019.02740

**Published:** 2019-11-28

**Authors:** Serge Ruden, Annika Rieder, Irina Chis Ster, Thomas Schwartz, Ralf Mikut, Kai Hilpert

**Affiliations:** ^1^Institute of Biological Interfaces, Karlsruhe Institute of Technology, Karlsruhe, Germany; ^2^Institute of Functional Interfaces, Karlsruhe Institute of Technology, Karlsruhe, Germany; ^3^Institute of Infection and Immunity, St George’s, University of London, London, United Kingdom; ^4^Institute for Automation and Applied Informatics, Karlsruhe Institute of Technology, Karlsruhe, Germany; ^5^Institute of Microstructure Technology, Karlsruhe Institute of Technology, Karlsruhe, Germany

**Keywords:** multidrug resistance, *Pseudomonas aeruginosa*, antimicrobial peptides, synergy, revive old antibiotics

## Abstract

With the rise of various multidrug-resistant (MDR) pathogenic bacteria, worldwide health care is under pressure to respond. Conventional antibiotics are failing and the development of novel classes and alternative strategies is a major priority. Antimicrobial peptides (AMPs) cannot only kill MDR bacteria, but also can be used synergistically with conventional antibiotics. We selected 30 short AMPs from different origins and measured their synergy in combination with polymyxin B, piperacillin, ceftazidime, cefepime, meropenem, imipenem, tetracycline, erythromycin, kanamycin, tobramycin, amikacin, gentamycin, and ciprofloxacin. In total, 403 unique combinations were tested against an MDR *Pseudomonas aeruginosa* isolate (PA910). As a measure of the synergistic effects, fractional inhibitory concentrations (FICs) were determined using microdilution assays with FICs ranges between 0.25 and 2. A high number of combinations between peptides and polymyxin B, erythromycin, and tetracycline were found to be synergistic. Novel variants of indolicidin also showed a high frequency in synergist interaction. Single amino acid substitutions within the peptides can have a very strong effect on the ability to synergize, making it possible to optimize future drugs toward synergistic interaction.

## Introduction

Among the most serious problems health care is facing is the increased number of infections caused by antibiotic-resistant bacteria, which can no longer be treated with previously active antimicrobial agents. In 2013, the World Health Organization (WHO) identified the development of antibiotic resistance as one of the major global threats to human society and recommended intensive monitoring to identify critical hot spots, in order to reduce transmission. The global spread of antibiotic resistance is one of the most interesting examples of biological evolution. It is highly relevant to both human and animal health and welfare with a direct impact on society. The primary cause of this situation is the excessive use of antibiotics (Berendonk et al., 2015). Although environmental bacteria are most probably the original source of many antibiotic resistance genes found in bacterial pathogens, the impact of nosocomial resistance and transmission has greatly increased over the last half century (Bush et al., 2011). The increased prevalence of antibiotic resistance in microbiota is due to four major factors: (i) the horizontal transfer of antibiotic-resistant genes, (ii) the assortment of resistant bacteria due to selective pressures from antimicrobial usage, (iii) the bacterial capability for gene mutation and recombination (e.g., presence of mutator determinants) (Wright and Sutherland, 2007; Davies and Davies, 2010; Cantón and Morosini, 2011), and (iv) the dissemination of resistant organisms from human and animal commensals that have adapted to antibiotic treatment of the host. Importantly, we cannot exclude the proliferation of antibiotic resistance due to the spread of resistant bacterial clones and their mobile genetic elements carrying antibiotic-resistance genes. This is due to spontaneous processes not necessarily linked with antibiotic resistance (Kohanski et al., 2010; Seiler and Berendonk, 2012; Baquero et al., 2013). Though the over-use of antibiotics may cause resistant populations, other biotic and abiotic factors including physicochemical conditions, pollution, induction of stress responses, bacterial adaptation, and phenotypic heterogeneity *inter alia* can enhance the effect of selective pressure. Evidence has shown that even in sub-inhibitory concentrations, antibiotics may still exert their impact on a microbial community (Andersson and Hughes, 2014).

The review of antimicrobial resistance in 2014 chaired by Jim O’Neill and initiated by the UK prime minister, published in 2016, estimates that by 2050 more people (10 million) will die each year from infections than the current number of people who die from cancer^1^. In order to maintain modern medical standards of care, novel antimicrobials urgently need to be discovered and developed, particularly those with novel modes of action, which are less likely to suffer cross-resistance to existing drugs. The WHO published a priority list in 2017^2^ of bacteria that are particularly problematic, in order to provide information and focus for drug development projects. Carbapenem-resistant *Pseudomonas aeruginosa* is in the highest category.

*Pseudomonas aeruginosa* is a rod-shaped, Gram-negative bacterium, which is naturally found in soil and water and therefore well adapted to humid environments. It is a clinically important, opportunistic pathogen, which may cause pneumonia and bacteremia in the elderly or immuno-compromised hosts, and is responsible for chronic, destructive lung disease in patients suffering from cystic fibrosis (Bhagirath et al., 2016). *P. aeruginosa* exhibits a higher intrinsic resistance to a number of antimicrobial agents compared to most other Gram-negative bacteria and is one of the ESKAPE pathogens (Yoneda et al., 2005). Additionally, rapid development of resistance to previously effective antimicrobials, such as fluoroquinolones, aminoglycosides, and polymyxins (Lupo et al., 2018), has been observed.

Unfortunately, there has been a significant reduction in the development of novel antimicrobial agents with many major pharmaceutical companies halting research in anti-infective agents. The fact there are very few new antimicrobial agents with a new mode of action increases the risk of a nightmare scenario where even “minor” infections could become serious health risks. As there is already only a limited number of anti-pseudomonal antibiotics and an increasing level of resistance, it is important to ascertain whether potential new antibiotics with different modes of action also synergize with “old” antimicrobials, especially for multidrug resistant (MDR) bacteria.

Antimicrobial peptides (AMPs), also called host defense peptides, represent a ubiquitous response in nature to overcome microbial infections and compete for an ecological niche (Hancock and Patrzykat, 2002). They are found in bacteria, fungi, plants, and animals. These peptides have emerged as central components of the innate defenses of both lower and higher organisms. The antimicrobial activities can include actions against Gram-negative and Gram-positive bacteria, including mycobacteria, fungi, and enveloped viruses (Hancock, 2001; Cole, 2003; Mania et al., 2010; Ramón-García et al., 2013; Silva et al., 2016). Of particular interest is their ability to kill MDR bacteria (Nuti et al., 2017). In addition, within the last two decades, it has become increasingly clear that various AMPs play a role in regulating the process of innate immunity. It has been reported that some AMPs can have direct and indirect chemotactic functions, regulate chemokine and cytokine production, and promote wound healing (Territo et al., 1989; Niyonsaba et al., 2002; Heilborn et al., 2003; Elssner et al., 2004; Di Nardo et al., 2007; Carretero et al., 2008). The direct antimicrobial activity has been studied on some examples and multiple bacterial targets of AMPs were discovered (Brogden, 2005), for example binding to RNA, DNA, or histones (Kobayashi et al., 2000; Hale and Hancock, 2007; Cho et al., 2009; Xie et al., 2011), blocking DNA-dependent enzymes (Marchand et al., 2006; Hilpert et al., 2010), blocking the synthesis of important outer membrane proteins (Carlsson et al., 1991), binding to the chaperon DnaK and the ribosome (Krizsan et al., 2015; Knappe et al., 2016; Mardirossian et al., 2018, 2019) and lipid 2 (de Leeuw et al., 2010; Schmitt et al., 2010). In addition, the effect of such peptides on blood components was studied (Yu et al., 2015). It is possible to use peptide libraries to screen and optimize AMPs (Ashby et al., 2017). With the already large number of AMPs and there various modes of action, a method for classifying such peptides according to their mode of action would be highly valuable. Recently, BioSAXS was used to develop this type of classification method (Von Gundlach et al., 2016, 2019).

Antimicrobial peptides will often be produced in abundance in one organism, for example, on the skin of amphibians (Yang et al., 2012). It has been shown that AMPs can synergize with each other to create the desired biological effect, including antibacterial, antitumor, and immunomodulatory action (Westerhoff et al., 1995; Yan and Hancock, 2001; Pöppel et al., 2015; Rahnamaeian et al., 2015; Hanson et al., 2019). Synergy between AMPs and conventional antibiotics has been studied *in vivo* and *in vitro* (Baindara et al., 2016; Otvos et al., 2018; Zharkova et al., 2019). Since synergy could potentially be an important feature for future drugs based on AMPs, we were interested in discovering the extent to which small changes in the peptide sequence influence the synergy with conventional antibiotics. In addition, we also investigated whether antibiotics with the same target/mode of action would synergize with a peptide in a similarly manner or not. In this study, we used MDR *P. aeruginosa* and designed AMPs.

## Materials and Methods

### Peptides and Antibiotics

The peptides used in this study were purchased from the Brain Research Centre at the University of British Columbia. Peptides were characterized and purified using high-performance liquid chromatography (HPLC); mass was confirmed by matrix-assisted laser desorption time of flight (MALDI-TOF) mass spectroscopy. The purity of all peptides was greater than 90%.

The antibiotics were purchased from VWR, except polymyxin B, which was purchased from Sigma-Aldrich.

### Minimal Inhibitory Concentration

We selected six different *P. aeruginosa* isolates that were described as MDR and had been isolated from clinical and municipal waste water (Schwartz et al., 2006). The minimal inhibitory concentration (MIC) was determined in a microdilution assay using Mueller-Hinton (MH) broth following a previously published protocol (Wiegand et al., 2008). Briefly, a twofold serial dilution of the antibiotics and peptides were prepared and added to a bacteria solution, resulting in 2–5 10^5^ CFU/ml. The microtiter plates (polypropylene, Corning) were incubated for 18 h at 37°C and MICs were taken visually.

### Fractional Inhibitory Concentration

The checkerboard assay was used to determine the fractional inhibitory concentrations (FICs), following the protocol described in Koneman’s Color Atlas and Textbook of Diagnostic Microbiology (Winn et al., 2006). Briefly, combinations of peptides and antibiotics were prepared in 96-well plates (polypropylene, Corning) in a twofold dilution series. After the addition of a log-phase bacterial inoculum of 2–5 × 10^5^ CFU/ml, plates were incubated at 37°C for 18 h. The FICs were determined by visual inspection and the effects of the combinations were determined using the FICs. The FIC was computed by adding two partial FIC values, FIC_A_—the MIC of drug A, tested in combination with drug B divided by the MIC of drug A, tested alone and FIC_B_—the MIC of drug B, tested in combination with drug A divided by the MIC of drug B, tested alone [FIC = FIC_A_ + FIC_B_ = (MIC_AB_/MIC_A_) + (MIC_BA_/MIC_B_), where MIC_A_ and MIC_B_ are the MICs of drugs A and B alone, respectively, and MIC_AB_ and MIC_BA_ are the MIC concentrations of the drugs in combination]. Here we use the European Committee for Antimicrobial Susceptibility Testing (EUCAST) definition, which is very similar to the definition provided by Odds, 2003, except Odds define additive effects when FIC values are between 0.5 and 1 (European Committee for Antimicrobial Susceptibility Testing [EUCAST] of the European Society of Clinical Microbiology and Infectious Diseases [ESCMID], 2000; Odds, 2003). The combination of peptide (drug A) and antibiotics (drug B) was defined as synergistic if the FIC was ≤ 0.5, indifferent if the FIC was > 0.5 but ≤ 4.0 and antagonistic if the FIC was > 4.0.

### Statistical Analysis

A statistical binary outcome has been defined according to two activity classes to analyze MIC values of the conventional antibiotics. Class 0 is denoted as sensitive and is defined by values below or equal to the EUCAST MIC breakpoint, while class 1 is denoted as resistant and is defined by values above the EUCAST MIC breakpoint. For the wild type (PA01), all tested antibiotics with breakpoints were sensitive. Cross-tabulating PA01 and any of PA910-PA253 would therefore not result in a proper 2 × 2 table, so a Fisher exact test would apply. However, given the biological context, it is possible to build a valid one-sample statistical test if we made the assumption that there is only one resistance in PA01wt and use a binomial exact test (with various approximations for 95% CI—only Wilson shown) for the null hypothesis H0: π = 0.1 against H1: π≠0.1 (Van Belle and Fisher, 2004). The assumption is that each resistance/sensitive value along columns is independent of each other.

A four-category ordinal response variable has been generated (from weak to strong in microbiological terms) to analyze the MIC values of the AMPs. The levels of outcome status have a natural ordering but the distances between adjacent levels are unknown. To understand how these levels associate with class, a natural approach is that of an ordinal logistic regression on class. The key assumption in ordinal regression is that the effects of any explanatory variables are consistent or proportional across the different thresholds; hence, this is usually termed proportional odds assumption (Van Belle and Fisher, 2004). A simple chi-squared statistical test would be limited to producing a *p*-value indicating the strength of the evidence against the null hypothesis of independency between this outcome and class activity. The advantage of an ordinal logistic regression is in that it numerically quantifies this potential association between the effect of class and this outcome derived from activity. All the analyses have been performed using *Stata Statistical Software: Release 15*. College Station, TX, United States: StataCorp LLC.

## Results

First, we determined the MIC values against a range of different antibiotics for six strains of *P. aeruginosa* which were described as MDR and had been isolated from clinical or municipal waste water, in comparison with a sensitive wild type strain (PAO1) (see Table 1; Schwartz et al., 2006). This confirmed that these strains are MDR. The response of wild-type strain PA01 toward the antibiotics was compared to the response of the six isolated MDR strains for statistical analysis. This response was classified into two groups, sensitive and resistant. The MIC EUCAST breakpoints were used to make the distinction between resistant (larger MIC than breakpoint) and sensitive (equal or lower MIC than breakpoint). The resulting *p*-values from these tests indicate strong evidence against the null, i.e., that all the proportions of resistance compared to wt of differs significantly from 0.1 [all the 95% confidence intervals (CIs) are well above 0.1] even after a conservative Bonferroni correction for multiple testing.

**TABLE 1:** MIC determination of conventional antibiotics against wild-type *P. aeruginosa* and MDR isolates (values in μM), at least three repeats for each value (*n* = 3).

**Antibiotic/strain**	**Break-point**	**PA01-wt**	**PA 910**	**PA 915**	**PA 919**	**PA 923**	**PA 927**	**PA253**
Polymyxin B	>1.7^∗^	0.125	0.125	0.25	0.125	0.25	0.25	0.125
Ciprofloxacin	>1.5	0.25	64	32	64	64	16	64
Tobramycin	>8.5	0.25–0.5	32	64	64	32	32	64
Gentamycin	>8.3	1	128	128–256	128	128	128	>256
Amikacin	>27.3	1	2–4	4	32	4	4	8–16
Imipenem	>13.4	2	>128	>128	16	> 128	>128	> 128
Meropenem	>20.8	2–4	>128	>128	>128	>128	>128	>128
Piperacillin	>30.9	4	64–128	>256	64	128	256	>256
Ceftazidime	>14.6	4	128	4	8	128	>128	>128
Tetracyclin	NA	8	64	16	32	>512	16	32
Cefepime	>16.6	8	64	32	16	64	128	>128
Kanamycin	NA	64	128–256	256–512	128	256	128	>512
Erythromycin	NA	128	128	64	128	256	16	64
Proportion of resistance compared to wt			0.8	0.7	0.8	0.8	0.8	0.8
*p*-Values			<0.000001	<0.000001	<0.000001	<0.000001	<0.000001	<0.000001
SE			0.13	0.14	0.13	0.13	0.13	0.13
95% Binomial CI			(0.44, 0.97)	(0.35, 0.93)	(0.44, 0.97)	(0.44, 0.97)	(0.44, 0.97)	(0.44, 0.97)
Wilson			(0.49, 0.94)	(0.40, 0.89)	(0.49, 0.94)	(0.49, 0.94)	(0.49, 0.94)	(0.49, 0.94)

*The breakpoints were taken from www.eucast.org/clinical_breakpoints/ and then converted to μM. ^∗^Breakpoint of colistin was used. Tetracycline, kanamycin, and erythromycin are not used in the clinic against *P. aeruginosa* and therefore exhibit no breakpoint. MIC values defined binary variables denoted as sensitive (below or equal breakpoint) and resistant (above breakpoint) for the statistical analysis. SE: standard error, CI: confidence interval.*

A set of short peptides between 9 and 13 amino acids in length were selected from published and ongoing unpublished work (Hilpert et al., 2005, 2006; Cherkasov et al., 2009; Mikut et al., 2016); LL37 was used as a comparison peptide. LL37 is a long (37mer) and helical peptide. The peptides are classified into four peptide classes: class A: Bac2A variants, class B: LL37, class C: RW-rich peptides, and class D: indolicidin variants. The MIC values of the peptides against the wild-type *P. aeruginosa* strain as well as three selected MDR variants of PA910, PA919, and PA253 were determined (Table 2). The majority of MIC values were equal or similar (plus/minus factor of two) between the wild-type and the MDR strains, with the highest change observed for indolicidin with an eightfold decrease in activity. The MIC values defined four classes according to their activity, where class 1 is the most active with MIC values of 1–2 μM, class 2 includes peptides with MIC values between 4 and 8 μM, class 3 includes peptides with MIC values between 16 and 32 μM, and the least active class includes peptides with MIC values larger than 32 μM. Statistical analysis suggested that peptide classes C and D are associated with stronger activity compared to class A (see Table 3). In addition, comparisons between strains showed *p*-values less than 0.001 only between wt PA01 and PA910.

**TABLE 2:** MIC values in μM of short AMPs (9-13mer) measured in triplicate (*n* = 3) against several isolates of MDR *P. aeruginosa*.

**Name**	**Amino acid sequence**	**Class**	**MIC against MDR isolates of *PA/*μM**			
			
			***PA01 wt***	***PA910***	***PA919***	***PA253***
Bac2a^1^	RLARIVVIRVAR-CONH_2_	A	16 (3)	32 (3)	16 (3)	32 (3)
G2^2^	RGARIVVIRVAR-CONH_2_	A	32 (3)	32 (3)	16 (3)	32 (3)
R2^2^	RRARIVVIRVAR-CONH_2_	A	32 (3)	32 (3)	16 (3)	32 (3)
W3^2^	RLWRIVVIRVAR-CONH_2_	A	16 (3)	32 (3)	32 (3)	32 (3)
R3^2^	RLRRIVVIRVAR-CONH_2_	A	16 (3)	32 (3)	16 (3)	16 (3)
W10^2^	RLARIVVIRWAR-CONH_2_	A	16 (3)	16 (3)	16 (3)	16 (3)
R11^2^	RLARIVVIRVRR-CONH_2_	A	16 (3)	64 (4)	32 (3)	64 (4)
Sub 3^2^	RRWRIVVIRVRR-CONH_2_	A	8 (2)	16 (3)	16 (3)	8 (2)
Sub 7	RLWRIVVIRVKR-CONH_2_	A	16 (3)	32 (3)	32 (3)	16 (3)
Bac034^3^	VRLRIRVAVIRA-CONH_2_	A	32 (3)	64 (4)	32 (3)	64 (4)
W3^3^	VRWRIRVAVIRA-CONH_2_	A	8 (2)	16 (3)	8 (2)	16 (3)
LL37	^##^	B	16 (3)	64 (4)	16 (3)	64 (4)
HHC-53^4^	FRRWWKWFK-CONH_2_	C	8 (2)	32 (3)	16 (3)	8 (2)
LOP1^5^	RWWRKIWKW-CONH_2_	C	2 (1)	8 (2)	2–4(2)	8 (2)
LOP2^5^	RRWWRWVVW-CONH_2_	C	4 (2)	8 (2)	2 (1)	8 (2)
LOP3^5^	KRRWRIWLV-CONH_2_	C	4 (2)	8 (2)	4 (2)	4–8(2)
LOP4^5^	RRWRVIVKW-CONH_2_	C	4 (2)	4 (2)	4 (2)	8 (2)
LOP5^5^	WKWLKKWIK-CONH_2_	C	4 (2)	8 (2)	8 (2)	8 (2)
Indolic.^6^	ILPWKWPWWPWRR-CONH_2_	D	8 (2)	32 (3)	32 (3)	64–128(4)
Indopt 1	FIKWKKRWWKKRT-CONH_2_	D	4 (2)	8 (2)	32 (3)	16 (3)
Indopt 2	FIKWRFRRWKKRT-CONH_2_	D	4 (2)	8 (2)	32 (3)	16 (3)
Indopt 3	FIKWRSRWWKKRT-CONH_2_	D	4 (2)	8 (2)	32 (3)	16 (3)
Indopt 4	FIKWRFRRWKKRK-CONH_2_	D	4 (2)	4–8(2)	16–32(3)	8 (2)
Indopt 5	FIKWKFRPWKKRT-CONH_2_	D	4 (2)	8 (2)	16–32(3)	16 (3)
Indopt 6	FIKRKSRWWKWRT-CONH_2_	D	4 (2)	8 (2)	32 (3)	8–16(3)
Indopt 7	ILKWKRKWWKWFR-CONH_2_	D	2 (1)	4 (2)	32 (3)	8 (2)
Indopt 8	ILKWKKGWWKWFR-CONH_2_	D	4 (2)	4 (2)	16 (3)	8 (2)
Indopt 9	ILKWKRKWWKWRR-CONH_2_	D	1 (1)	2 (1)	16 (3)	4 (2)
Indopt 10	ILKWKIFKWKWFR-CONH_2_	D	2 (1)	4 (2)	32 (3)	8–16(3)
Indopt 11	ILKWKTKWWKWFR-CONH_2_	D	2 (1)	4 (2)	16 (3)	4 (2)
Indopt 12	ILKWKMFKWKWFR-CONH_2_	D	2 (1)	4 (2)	16 (3)	16 (3)

*MIC values of all Indopt variants were measured in 1/8 MHb, while all other were measured in full MHb. A 37mer human AMP was included as comparison (LL37). ^##^The sequence of the peptide LL-37: LLGDFFRKSKEKIGKEFKRIVQRIKDFLRNLVPRTES-COOH_2_. ^1^(Wu and Hancock, 1999a), ^2^(Hilpert et al., 2005), ^3^(Hilpert et al., 2006), ^4^(Cherkasov et al., 2009), ^5^(Mikut et al., 2016), ^6^(Selsted et al., 1992). The MIC values were compiled into four classes according to their activity, where class 1 is the most active with MIC values of 1–2 μM, class 2 includes peptides with MIC values between 4 and 8 μM, class 3 includes peptides with MIC values between 16 and 32 μM, and the least active class includes peptides with MIC values larger than 32 μM. The class values are shown in parentheses after the MIC values, e.g., 32(3) means MIC value 32 μM belongs to class 3.*

**TABLE 3:** Statistical analysis of activity classes based on MIC values given in Table 2.

**Outcome level/Class**	**Class A**	**Class C**	**Class D**	**Total**

1 (Strong)	0	2	6	8
2 (Good)	4	20	24	48
3 (Medium)	36	2	21	59
4 (Weak)	4	0	1	5
Total	**44**	**24**	**52**	**120**

**Comparison: classes**	**Odds ratio**	***P*-value**	**95%CI—L**	**95%CI—H**

C vs. A	47.56225	<0.001	13.22247	171.0852
D vs. A	15.43157	<0.001	5.267699	45.20632
C vs. D	3.08214	0.027	1.134687	8.371993

**Comparison: strains**	**PA01wt**	**PA910**	**PA919**	**PA253**

PA01wt		*p* < 0.001	*p* = 0.308	*p* = 0.013
PA910			*p* = 0.721	*p* = 0.001
PA919				*p* = 0.119
PA253				

Strain PA 910, which is only sensitive to polymyxin B and amikacin, was selected to perform a synergy study combining 31 AMPs with 12 antibiotics and one lipopeptide (polymyxin B) resulting in 403 unique combinations (see Table 4).

**TABLE 4:** Fractional inhibitory concentrations (FICs) of short AMPs, 9-13mers, and different antibiotics were determined against an MDR isolate of *P. aeruginosa* (PA 910).

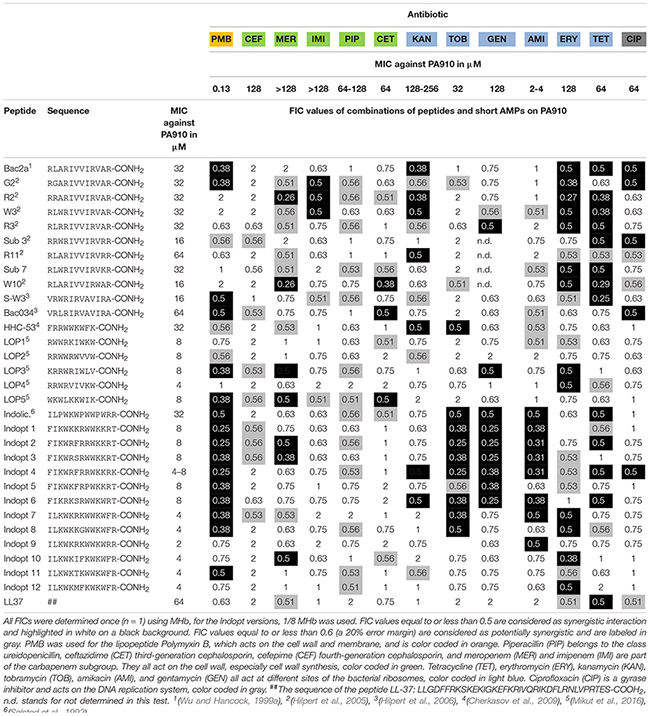		

**FIGURE 1:**
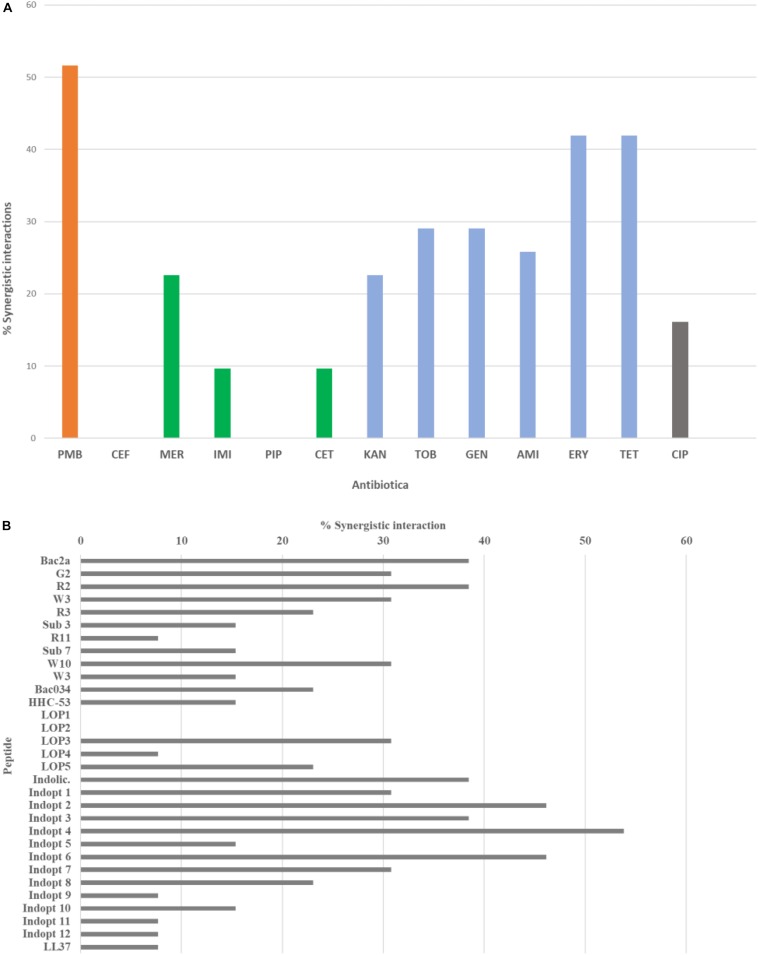
The maximum number of possible interactions was set to 100% and the percentage of synergistic interaction was calculated for **(A)** conventional antibiotics and **(B)** short antimicrobial peptides. For color codes and abbreviations, see Table 4.

The synergistic effects of combining short AMPs and conventional antibiotics resulted in a complex pattern. There were peptides that show no synergistic effect, while others showed a variety, similar to the tested antibiotics (see Figures 1A,B).

For further statistical analysis, FIC values were transposed into two classes, class 0 with FIC values larger than 0.5 and class 1 FIC values equal or less than 0.5. *P*-values for the null hypothesis that FIC values are similar were calculated comparing the antibiotics and between peptide classes (see Table 5).

**TABLE 5:** Top section: *p*-values for the null hypothesis that FIC values of all combinations are similar. ^∗^For PIP and CEF, binomial tests were carried for the null hypothesis that the proportion equals 1/30 = 0.0333. Bottom section: *p*-value calculated for three different peptide classes. The total sum of counts for synergy are given for each class with regard to each antibiotic.

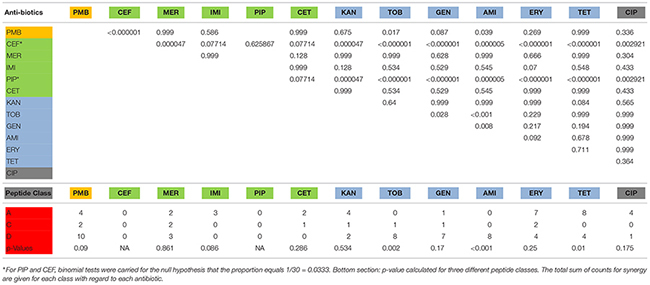

Selected peptide–antibiotic combinations were tested three times to confirm first findings (see Table 6).

**TABLE 6:** Mean values (at least three measurements, *n* = 3) and standard deviations (values in brackets), of fractional inhibitory concentrations (FICs) of selected short AMPs, 9-13mers, and different antibiotics were determined against an MDR isolate of *P. aeruginosa* (PA 910).

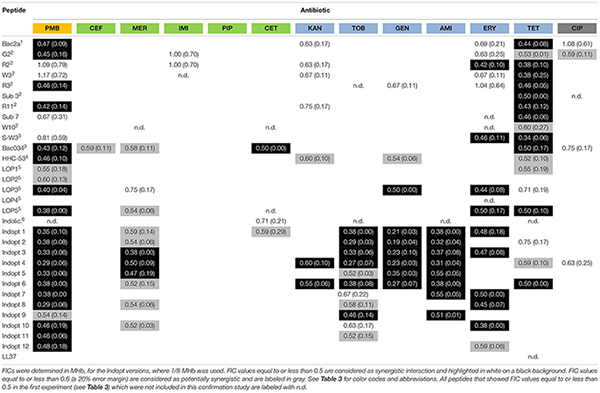

To test whether the observed synergy was dependent on the selected strain or not, two additional MDR PA isolates (PA253 and PA919) were used to determine the FICs of the selected peptides applied with polymyxin B (see Table 7).

**TABLE 7:** FIC values of selected peptides for three different MDR *P. aeruginosa* strains against polymyxin B.

**Name**	**Sequence**	**MDR-PA910**	**MDR-PA253**	**MDR-PA919**
Indopt 4	FIKWRFRRWKKRK-CONH_2_	0.29	0.29	0.38
Indopt 8	ILKWKKGWWKWFR-CONH_2_	0.29	0.31	0.38
Indopt 3	FIKWRSRWWKKRT-CONH_2_	0.33	0.19	0.38
Indopt 5	FIKWKFRPWKKRT-CONH_2_	0.33	0.13	0.38
Indopt 1	FIKWKKRWWKKRT-CONH_2_	0.35	0.38	0.38
Indopt 2	FIKWRFRRWKKRT-CONH_2_	0.38	0.31	0.38
Indopt 6	FIKRKSRWWKWRT-CONH_2_	0.38	0.26	0.38
Indopt 7	ILKWKRKWWKWFR-CONH_2_	0.38	0.52	0.29
LOP5	WKWLKKWIK-CONH_2_	0.38	0.25	0.38
LOP3	KRRWRIWLV-CONH_2_	0.40	0.31	0.56
R11	RLARIVVIRVRR-CONH_2_	0.42	0.56	0.56
Bac034	VRLRIRVAVIRA-CONH_2_	0.43	0.38	0.38
HHC53	FRRWWKWFK-CONH_2_	0.46	0.63	0.50
Indopt 10	ILKWKIFKWKWFR-CONH_2_	0.46	0.50	0.38
Indopt 11	ILKWKTKWWKWFR-CONH_2_	0.46	0.50	0.29
R3	RLRRIVVIRVAR-CONH_2_	0.46	0.50	0.63
Indopt 12	ILKWKMFKWKWFR-CONH_2_	0.48	0.50	0.27
Indopt 9	ILKWKRKWWKWRR-CONH_2_	0.54	0.63	0.50
Sub7	RLWRIVVIRVKR-CONH_2_	0.67	0.50	0.50

*Values from MDR-PA910 are mean values from at least three different measurements; values from MDR-PA253 and MDR-PA919 are measured once. The table is sorted according to the values from MDR-PA910, from low to high.*

## Discussion

*Pseudomonas aeruginosa* is ranked among the top five organisms causing bloodstream, urinary tract, pulmonary, surgical site, and soft tissue infections in patients in intensive care units (Veesenmeyer et al., 2009). The bacterium is widely distributed in the environment, as it can utilize a wide range of materials for its nutrients, while only requiring a limited amount of nutrients to survive (Abdelraouf et al., 2011). The current treatment regimen for MDR cases is limited to the last resort antibiotic Colistin (Hachem et al., 2007; Sabuda et al., 2008). Alarmingly, however, *P. aeruginosa* resistance to colistin has been reported (Goli et al., 2016). The situation is becoming increasingly disconcerting, and the WHO has declared it a “critical priority pathogen,” on which research and development of novel antibiotics should be focused (Tacconelli et al., 2018).

We confirmed the previously described multidrug resistance of *P. aeruginosa* strains which were isolated in clinical and municipal waste water (see Table 1; Schwartz et al., 2006). Statistical tests showed very low *p*-values demonstrating statistically significant differences between the susceptible wt PA01 strain and the MDR strains. It has been reported that AMPs show synergy with conventional antibiotics both in planktonic and biofilm growth (Giacometti et al., 2000; Jorge et al., 2017). Here we studied whether a set of short AMPs which we developed in previous projects can synergize with antibiotics in order to revive them. We also included polymyxin B, since treatment failures with monotherapy of polymyxins are reportedly increasing, making it an urgent candidate for use with synergistic agents. Clinical trials investigating colistin alone versus colistin in combination with meropenem are currently underway (ClinicalTrials.gov IDs NCT01732250 and NCT01597973) (Lenhard et al., 2016).

Bactenecin (RLCRIVVIRVCR-CONH_2_) is a cyclic dodecapeptide found in bovine neutrophils. It is stored in granules reaching concentrations of about 12 mg/ml and is produced as a 155-mer precursor polypeptide (Romeo et al., 1988; Storici et al., 1992). The peptide belongs to the cathelicidine family. Using NMR spectroscopy, an antiparallel β-sheet structure stabilized by a single disulfide bond was detected (Raj et al., 2000). Synthetically produced bactenecin demonstrated modest antibacterial activities against Gram-negative and Gram-positive pathogens (Romeo et al., 1988; Gallis et al., 1989; Wu and Hancock, 1999b). Linear variants were produced, for example, Bac2A (RLARIVVIRVAR-CONH_2_) which showed similar antibacterial activity and an improved toxicological profile (Wu and Hancock, 1999a). The cyclic peptide bactenecin showed weak activity against artificial membranes, and the cytoplasmatic membrane of *Escherichia coli* was also only mildly disrupted at MIC concentrations (Wu et al., 1999). In the case of *Burkholderia pseudomallei*, bactenecin showed a strong binding with LPS, weak outer membrane permeabilization, and medium activity monitoring inner membrane disruption (Madhongsa et al., 2013). In the case of tested linear variants, they caused a faster and almost 100% depolarization in *E. coli*, showing a distinctive different pattern. In the case of *Staphylococcus aureus*, similar results were obtained for linear bactenecin variants (Wu et al., 1999; Hilpert et al., 2006). Linear bactenecin variants showed a random structure in water, but a β-structure in contact with liposomes (Hilpert et al., 2006). A complete substitution analysis of the linear peptide Bac2A was performed using spot synthesis and a luminescent variant of *P. aeruginosa* (Hilpert et al., 2005, 2007; Hilpert and Hancock, 2007). This information led to various variants that differ only slightly in sequence but strongly in activity. In addition, scrambled variants were created and one of them further investigated by substation analysis (Hilpert et al., 2006). Bac2A and other variants were also shown to interact with ATP and can inhibit luciferase, DnaK, and DNA polymerase (Hilpert et al., 2010). The precise mechanism via which bactenecin, Bac2A, and other linear variants kill the bacteria is still unresolved but data indicate multiple mechanisms. The peptide Bac2A showed synergistic interactions with five different antibiotics, PMB, KAN, ERY, TET, and CIP. The exchange of a single amino acid on position 2 or 3 (G2, R2, and W3) leads to additional synergy with IMI. The single amino substitution R2 and W10 lead to a strong additional synergistic effect with MER. In contrast, the single amino acid substitution R11 reduced the synergistic interaction to only one antibiotic, KAN. The antibiotics CEF, PIP, TOB, and AMI did not exhibit any synergy with this class of peptides.

The 9mer peptides HHC-53 and LOP1-5 are *in silico* predicted peptides using different software (Cherkasov et al., 2009; Mikut et al., 2016). The specific peptide HHC-53 was shown to be effective in an invasive *S. aureus* mouse model. The mode of action is unknown. Three of these 9mer peptides do not show any synergy with the tested antibiotics. The peptide LOP3 exhibits the most synergistic interaction in this class, which includes PMB, MER, GEN, and ERY.

The 13mer peptide indolicidin (ILPWKWPWWPWRR-CONH_2_) is, like bactenecin, a bovine peptide and also belongs to the cathelicidin family. It is present in the cytoplasmic granules of neutrophils (Selsted et al., 1992). The NMR structure reveals that indolicidin forms an extended boat-like structure (Rozek et al., 2000). The peptide is modestly active against various Gram-positive and Gram-negative bacteria, but exhibits high hemolytic activity and cytotoxicity (Selsted et al., 1992; Ahmad et al., 1995). In Gram-negative bacteria, indolicidin interacts with surface-exposed lipopolysaccharides (LPS), resulting in a self-promoted uptake across the outer membrane, followed by channel formation in the cytoplasmic membrane, leading to cell death (Falla et al., 1996). Indolicidin has rather weak membrane permeabilization characteristics (Wu et al., 1999). Similarly to Bac2A, indolicidin seems to have multiple modes of action; it interacts with ATP and can inhibit ATP dependent enzymes, inhibits DNA/RNA synthesis, and inhibits protein synthesis (Subbalakshmi and Sitaram, 1998; Hilpert et al., 2010). In this study, optimized indolicidin variants are used in addition to indolicidin itself. Variants Indopt1-6 show a similar pattern compared to indolicidin itself. Indopt 2 and 3 also show synergy with MER; Indopt 4 shows additional synergy to KAN and CIP. Indopt 5 shows only two synergistic interactions, again showing that small changes in the sequence can have a strong effect on the synergy. Indopt 7-12 show only very few synergistic interactions.

The FICs between combinations of antibiotics and peptides show several synergistic effects if antibiotics are used in tandem with short AMPs. In general, the beta-lactams and beta-lactam-like antibiotics show the lowest amount of synergy, along with ciprofloxacin, a gyrase inhibitor, which also showed a rather low amount of synergy. Cefepime and piperacillin showed no detectable synergy at all. However, antibiotics acting on the ribosome show a higher amount of synergy, with the highest proportion of synergy observed in the use of polymyxin B, which acts on the cell wall and cell membrane. The majority of the results were confirmed by three independent measurements of the selected combinations. Some combinations, however, showed larger FIC values than determined in the first screen, thus proving the importance of verifying FIC data (Hsieh et al., 1993).

Polymyxin B binds to the lipid A portion of the LPS, to replace cationic ions such as Ca^2+^ and Mg^2+^ from the LPS layer (Morrison and Jacobs, 1976). This process destabilizes the LPS layer, leading to permeability changes and consequently to a “self-promoted uptake” (Hancock, 1997; Hermsen et al., 2003). This process destabilizes the membrane and allows molecules to pass through the membrane in both directions. It has been shown that polymyxin B synergizes with antibiotics (Elemam et al., 2010; Zusman et al., 2013; Abdul Rahim et al., 2015) as well with AMPs (Giacometti et al., 2000; van der Linden et al., 2009; Draper et al., 2013). The majority of short cationic peptides used in these studies also showed synergistic interaction with polymyxin B (Table 4). This synergy was verified for two additional strains (Table 5). Small changes in the sequence of Bac2A lead to a loss in synergy, which is especially pronounced when a tryptophan residue is introduced, for example, W3, Sub3, W10, and S-W3. The data suggest that an additional tryptophan residue might anchor the peptide more in the membrane and consequently does not support synergy with polymyxin B.

Cefepime is a fourth-generation cephalosporin antibiotic that has an extended spectrum of activity against Gram-positive and Gram-negative bacteria and is more stable compared to third-generation agents (Wynd and Paladino, 1996). Cephalosporins are bactericidal that disrupts the synthesis of the peptidoglycan layer of the bacterial cell walls by blocking transpeptidases known as penicillin-binding proteins (PBPs) (Klein and Cunha, 1995). There was no synergistic effect observed, indicating that the peptides did not improved access to target sites or that they interfered with the lactamases. Ceftazidime is a third-generation cephalosporin antibiotic targeting PBPs. Similar to cefepime, there were no synergistic combinations determined with the exception of Bac034, W10, and LOP5. Meropenem, a carbapenem-type beta-lactam antibiotic active against Gram-positive and Gram-negative bacteria, blocks PBPs and shows a bactericidal activity (Blumer, 1997). In contrast to cefepime, a range of short AMPs showed synergistic interaction. Imipenem on the other hand, another carbapenem-type beta-lactam with the same mode of action, did show three synergistic interactions; however, in the confirmation experiment (Table 4), these were not verified (Park and Parker, 1986). We conclude that synergy is caused by a meropenem-specific feature, for example, increasing the uptake rate for this molecule. Piperacillin is a penicillin beta-lactam antibiotic used clinically mainly for Gram-negative organisms and demonstrates bactericidal activity as a result of the inhibition of cell wall synthesis by binding to PBPs. Piperacillin is stable against hydrolysis by a variety of beta-lactamases, including penicillinases, cephalosporinases, and extended spectrum beta-lactamases (Eliopoulos and Moellering, 1982). No synergistic combinations were observed for piperacillin with the short AMPs tested.

Kanamycin (kanamycin A) belongs to the aminoglycoside class and is a natural compound found in *Streptomyces kanamyceticus* (de Lima Procópio et al., 2012). Aminoglycosides bind to the 30S subunit of the ribosome: most binding occurs on the 16 srRNA of the bacteria leading to a bactericidal action (Walter et al., 1999). It shows broad-spectrum activity against Gram-negative bacteria and some activity against Gram-positive. Of all the aminoglycosides, kanamycin shows the least activity and the least confirmed synergy. Tobramycin is produced in *Streptomyces tenebrarius* and belongs to the class of aminoglycoside antibiotics. It binds irreversibly to the 30S ribosomal subunit and shows a broad-spectrum activity, especially effective against *P. aeruginosa*^3^. Gentamicin is an aminoglycoside antibiotic that is produced by *Micromonospora purpurea* and acts on the 30S subunit. It is highly active against Gram-negative bacteria and shows activity against some Gram-positive bacteria (Daniels et al., 1975). Amikacin is a semi-synthetic antibiotic based on kanamycin A, both members of the class aminoglycosides, which binds to the 30S subunit and 16 srRNA. Amikacin demonstrates a broad-spectrum activity toward Gram-negative bacteria, including pseudomonades, and has some effects on Gram-positive bacteria, including *S. aureus* (Ristuccia and Cunha, 1985). Indolicidin and Indopt1, 2, 3, 4, and 6 show strong synergistic interactions with tobramycin, gentamycin, and amikacin. This observation is in accordance with research published by Boehr et al. (2003), showing that indolicidin and analogs (differ from the ones in this study) are able to inhibit aminoglycoside phosphotransferase and aminoglycosides acetyltransferase. Through the inhibition of these enzymes, the most effective resistance mechanism in the bacteria can be weakened and activity of the antibiotics gained (Boehr et al., 2003). Based on these findings, a further optimization of Indopt peptides could lead to the creation of more potent aminoglycoside phosphotransferase and aminoglycosides acetyltransferase inhibitors that can be used for a combination therapy to overcome aminoglycosides resistance.

Erythromycin is a macrolide antibiotic produced by *Saccharopolyspora erythraea* and reversibly binds to the 50S subunit of the bacterial ribosome^4^. It is active against Gram-negative and Gram-positive bacteria. Tetracycline is a naturally produced antibiotic by *Streptomyces aureofaciens* and binds reversibly to the 30S subunit as well as to some extent the 50S subunit, with potential influence over the bacterial membrane^5^. Tetracycline belongs to the class of tetracyclines and is a broad-spectrum antibiotic with activity against Gram-positive and Gram-negative bacteria. The synergy patterns for these two classes appear different from the aminoglycosides. A broader range of peptides can synergize with these antibiotics, for example, Bac2A variants, 9mer variants, and the indolicidin variants.

Ciprofloxacin is a synthetic antibiotic belonging to the fluoroquinolones and is an inhibitor of the bacterial topoisomerase II (DNA gyrase) and topoisomerase IV. Ciprofloxacin is a broad-spectrum antibiotic with a wide range of Gram-positive and Gram-negative bacteria^6^. Only a few peptides, Bac2A, G2, Sub3, and Indopt4, show synergy, all at 0.5, and confirmation studies showed even higher values for the selected combinations.

The perception of peptides as drugs is only slowly changing, despite the fact that in various cases, their value has been demonstrated. Over the last 30 years, more than 100 peptide-based drugs have been released for clinical use against a variety of pathologies including: diabetes, cancer, obesity, cardiovascular disease, inflammation, and osteoporosis; generating a net revenue of over US$40 billion annually (Mäde et al., 2014). Peptide-based drugs have multiple advantages, such as ease of synthesis and scalability as well as known degradation pathways leading to non-toxic by-products. As a result, in the years from 2016 to 2024, the growth in the development of peptide-based drugs is expected to rise by 9.1% and exceed US$70 billion in revenue by the end of 2019 (Lee et al., 2019).

Delivery of peptides are usually by injection, intravenous (IV), intramuscular (IM), by a catheter, by inhalation, or by subdermal osmotic pumps. Oral bioavailability is usually rather low. When further investigating AMPs for synergistic interaction in mouse models, we believe that IV delivery or the usage of an osmotic pump will be most relevant to draw conclusions for application in humans. Peptides HHC-53 and HHC-10 (9mer peptide from same study) were used in an invasive *S. aureus* mouse model and application of peptides was done by intraperitoneal (IP) for both peptides and IV for HHC-10. Recently, Knappe et al. (2019) have shown that the use of an osmotic pump leads to stable plasma levels that in consequence enhanced the survival rate compared to IV and IM administration. In our opinion, further *in vivo* studies for synergy should be performed when drug formulation and delivery is optimized. The oral availability of most antibiotics described here will ease the study of peptide–antibiotic synergy *in vivo* since two different delivery modes can be applied, controlling the required concentrations.

## Conclusion

In summary, our data show that small AMPs that can kill MDR bacteria, in this case MDR *P. aeruginosa*, are able to synergize with conventional antibiotics despite the fact that they are no longer effective. We believe that this shows the potential to develop these molecules not only as mono-therapeutic agents, but also as part of a combination therapy with the conventional antibiotics in order to reuse antibiotics via this synergistic approach with AMPs. This may be an alternative method for dealing with the resistance crisis. The data also show that small changes in the sequence can have quite dramatic effects on synergy, making it possible to optimize novel drugs toward desired synergistic effects.

## Author Contributions

SR performed the majority of laboratory work. TS supported laboratory work, provided MDR *Pseudomonas aeruginosa* strains, and wrote a part of the manuscript. AR performed laboratory work and supported the design and test of the checkerboard assay. RM supported the analysis of data and wrote a part of the manuscript. KH conceived and designed the experiments, wrote different sections of the manuscript, and collated all parts in a uniform style. IC defined the statistical outcomes, performed the statistical analyses, and wrote the corresponding sections of the manuscript.

## Conflict of Interest

The authors declare that some of the peptides used in this study are protected by patents. KH is the Founder and Director of TiKa Diagnostics Ltd. The remaining authors declare that the research was conducted in the absence of any commercial or financial relationships that could be construed as a potential conflict of interest.

## Footnotes

1 https://amr-review.org/Publications.html

2 http://www.who.int/news-room/detail/27-02-2017-who-publishes-list-of-bacteria-for-which-new-antibiotics-are-urgently-needed

3 https://www.drugbank.ca/drugs/DB00684

4 https://www.drugbank.ca/drugs/DB00199

5 https://www.drugbank.ca/drugs/DB00759

6 https://www.drugbank.ca/drugs/DB00199
